# Identification of diagnostic hub genes related to energy metabolism in idiopathic pulmonary fibrosis

**DOI:** 10.3389/fmolb.2025.1596364

**Published:** 2025-06-26

**Authors:** S. Zhao, B. C. Sun, N. Liu, R. Huo, L. S. Liu, J. P. Wang, C. Y. Fang

**Affiliations:** ^1^ College of Traditional Chinese Medicine, Hebei University of Chinese Medicine, Shijiazhuang, Hebei, China; ^2^ College of Traditional Chinese Medicine, North China University of Science and Technology, Tangshan, Hebei, China; ^3^ Laboratory of Lung Disease Research of Integrated Traditional Chinese and Western Medicine, Shijiazhuang, Hebei, China

**Keywords:** IPF (idiopathic pulmonary fibrosis), hub genes, energy matebolism, GEO, DGE analysis

## Abstract

**Background:**

Idiopathic pulmonary fibrosis (IPF) is a chronic and progressive lung disease that worsens over time, culminating in respiratory failure. Emerging evidence implicates dysregulated energy metabolism in driving fibroblast activation and extracellular matrix remodeling during IPF pathogenesis. To systematically investigate metabolic reprogramming mechanisms, we performed integrated bioinformatics analyses focusing on energy metabolism-related differentially expressed genes (EMRDEGs) and their regulatory networks in fibrotic remodeling.

**Methods:**

Differentially Expressed Genes (DEGs) were identified by accessing datasets GSE242063 and GSE110147 from the GEO database. Energy metabolism-related genes (EMRGs) were extracted from GeneCards, followed by Venn diagram analysis to obtain EMRDEGs. Subsequent analyses included functional enrichment (GO/KEGG), protein-protein interaction network, and mRNA-miRNA, mRNA-transcription factor interaction networks. Immune infiltration analyses, including the CIBERSORT algorithm, and single-sample gene set enrichment analysis (ssGSEA), were subsequently conducted.

**Results:**

We identified 12 EMRDEGs and eight hub genes (*ACSL1*, *CEBPD*, *CFH*, *HMGCS1*, *IL6*, *SOCS3*, *TLR2*, and *UCP2*). Regulatory network analysis revealed *HMGCS1* as a novel IPF-associated gene interacting with PPARα signaling, while *SOCS3* coordinated multiple hub genes (*IL6*, *CEBPD*, *UCP2*, and *CFH*) through FOXA1/2-mediated transcriptional regulation alongside JAK/STAT3 pathway suppression. Immune profiling demonstrated significant hub gene-immune cell correlations, particularly neutrophil-mediated differential gene expression and microenvironment remodeling.

**Conclusion:**

The core EMRDEGs (*HMGCS1* and *SOCS3*) and prioritized pathways (PPARα signaling, FOXA networks, JAK/STAT3 suppression) elucidate metabolic reprogramming mechanisms in fibrotic progression. These molecular signatures provide novel clinical biomarkers for IPF diagnosis.

## 1 Introduction

IPF is a chronic and progressive fibrotic lung disease that eventually leads to a decline in respiratory function and death (Raghu et al., 2022). IPF is a common interstitial lung disease affecting elderly patients over 65 years old, with survivors having a median lifespan of 2–3 years. The incidence ranges from three to nine cases per 100,000 per year (Kreuter et al., 2019; Podolanczuk et al., 2023). Current studies on IPF suggest that it is caused by repetitive damage to the lung epithelium, combined with myofibroblast activation and immune responses, which leads to dysregulated remodelling of lung tissue, culminating in self-perpetuating fibrosis (Koudstaal and Wijsenbeek, 2023). Despite recent advances in therapeutic strategies, treatment options remain limited; only nintedanib and pirfenidone have been approved for IPF treatment. Both drugs focus primarily on slowing disease progression but are accompanied by numerous side effects (Chianese et al., 2024; Kou et al., 2024). These unresolved challenges highlight the imperative to define core molecular events in IPF pathogenesis, with a focus on pathways that sustain fibrotic progression, thereby guiding future investigations into disease-modifying strategies.

While the pathogenesis of IPF remains unknown, studies have indicated that dysregulation of energy metabolism plays a critical role in the development and progression of fibrotic diseases (Gibb et al., 2020; Yakupova et al., 2021). The pathological characteristics are primarily marked by immune cell infiltration within the lungs, extracellular matrix deposition, fibroproliferative changes, and destruction of alveolar architecture (Heukels et al., 2019; Lu et al., 2025). Energy metabolism pathways, including glycolysis, lipid metabolism, and the tricarboxylic acid cycle, have been identified as contributing factors to the development of IPF. Metabolomics studies have revealed that free fatty acids exhibit abnormal accumulation in the lung tissue of IPF patients (Yan et al., 2017). The sphingolipid metabolic pathway is downregulated, whereas the arginine metabolic pathway is upregulated. Concurrently, glycolysis, mitochondrial β-oxidation, and the tricarboxylic acid cycle are disrupted, and significant alterations are observed in glutamate metabolism and other related pathways (Kang et al., 2016; Zhao et al., 2017). Free fatty acids can cooperate with transforming growth factor-β (TGF-β) to induce the activation of pulmonary myofibroblasts (Wygrecka et al., 2023). The levels of advanced glycation end products (AGEs) are significantly elevated in the serum and lung tissues of patients with IPF (Machahua et al., 2016; Yan et al., 2017). These AGEs can induce the upregulation of cytokines, including TGF-β1, tumor necrosis factor-α (TNF-α), and interleukin-8 (IL-8), thereby promoting the expression of type I and type III collagen (Serban et al., 2016). Various studies have revealed that there were changes in pathways of energy metabolism during remodeling of lung structural and metabolic abnormalities serve as key contributors to the activation of inflammatory factors. Therefore, the application of bioinformatics technology to identify key genes associated with energy metabolism in IPF and to explore their correlation with immune infiltration may facilitate a deeper understanding of the mechanisms underlying abnormal energy metabolism in IPF.

In summary, IPF remains a fatal disease lacking timely diagnosis and effective therapies. By integrating bioinformatics analysis, our study identifies energy metabolism-related DEGs (EMRDEGs) and hub genes as potential biomarkers, while exploring their immune infiltration correlations. These findings establish a molecular framework to dissect metabolic dysregulation in fibrotic remodeling, aiming to advance mechanistic insights and prioritize candidate targets for IPF.

## 2 Materials and methods

### 2.1 Data collection

The GEOquery package (Version 2.70.0) was utilized to download two datasets: GSE24206 (https://www.ncbi.nlm.nih.gov/geo/query/acc.cgi?acc=GSE24206) and GSE110147 (https://www.ncbi.nlm.nih.gov/geo/query/acc.cgi?acc=GSE110147) from the Gene Expression Omnibus database (GEO) (Barrett et al., 2007; Edgar, 2002). Both of them were from *Homo sapiens* (Davis and Meltzer, 2007). GSE24206 was performed on the GPL570 platform and contained 17 cases of IPF patients’ lung tissues and six cases of healthy adults’ surgical biopsy lung tissues. GSE110147 was performed on the GPL6244 platform and contained 22 cases of fresh frozen lung samples from IPF patients, ten cases with non-specific interstitial pneumonia (NSIP), 5 cases with mixed IPF-NSIP undergoing lung transplantation, and 11 cases of normal lung tissue samples obtained from tissue flanking lung cancer resections. These datasets were selected for their well-annotated transcriptomic profiles of IPF and control lung tissues, complemented by heterogeneous technical platforms and sample sources to strengthen analytical robustness and generalizability while controlling for platform-specific biases. All samples in GSE24206 and GSE110147 were selected for subsequent analysis. The specific grouping of datasets’ information is shown in Supplementary Table S1. The bioinformatic workflow is presented in Supplementary Figure S1.

### 2.2 Identification of differentially expressed genes related to energy metabolism

GeneCards (https://www.genecards.org/) (Safran et al., 2010) provides comprehensive details about human genes. We used ‘Energy Metabolism’ as the input keywords and selected ‘Protein Coding’ and a relevance score >1 as the selection criteria during the search process. Consequently, a total of 1,089 EMRGs were obtained. The details can be found in Supplementary Table S2.

To identify potential diagnostic and therapeutic targets and pathways of differentially expressed genes (DEGs) in Idiopathic Pulmonary Fibrosis (IPF), we utilized the limma package (Version 3.58.1) (Ritchie et al., 2015) for the analysis of IPF datasets (GSE24206 and GSE110147). This provided us with DEGs between two groups (IPF/Control). DEGs that displayed |logFC| > 1 and P < 0.05 were then selected and a Venn diagram was designed to arrive at the EMRDEGs. The results from this analysis were visualized using a volcano plot and heatmap via the ggplot2 package (Version 3.4.4), alongside the pheatmap package (Version 1.0.12).

### 2.3 Functional enrichment analysis of energy metabolism-related differentially expressed genes

The Gene Ontology (GO) enrichment analysis (The Gene Ontology Consortium, 2015) is widely used in the studies of systems biology to characterize extensive sets of genes. These include the biological process (BP), cellular component (CC), and molecular function (MF). The Kyoto Encyclopedia of Genes and Genomes (KEGG) pathway enrichment analysis (Kanehisa and Goto, 2000) serves as a knowledge database comprising genomic information, biological pathways, diseases, and drugs. Utilizing the clusterProfiler package (Version 4.10.0) (Yu et al., 2012), we conducted GO and KEGG enrichment analysis of EMRDEGs.

### 2.4 Gene set enrichment analysis (GSEA)

GSEA (Subramanian et al., 2005) is often executed to evaluate changes in BP activity and pathways in the samples of datasets. In our research, all DEGs in GSE24206 and GSE110147 were partitioned into two groups based on their positive and negative logFC values. Subsequently, the clusterProfiler package was implemented to perform GSEA with 2022 as seeds and 1,000 as the calculation number. Each gene set contained at least ten genes and a maximum of 500 genes. We downloaded the gene set “c2.cp.v2022.1. Hs.symbols. [All Canonical Pathways](3,050)” from the Molecular Signatures Database (MSigDB v2022.1. Hs) (Liberzon et al., 2015). The thresholds for significant enrichment were set at P < 0.05 and FDR <0.05.

### 2.5 Protein-protein interaction (PPI) network

The PPI network (Majeed and Mukhtar, 2023) comprises individual proteins that interact with each other, participating in biological signalling, gene expression regulation, and various essential processes like energy metabolism. The STRING database (https://string-db.org/) (Szklarczyk et al., 2023) captures PPI in both physical interactions and functional contexts. This study constructed a PPI network of EMRDEGs using the STRING database and visualized the network with Cytoscape (Version 3.9.1) (Shannon et al., 2003). All connected nodes of EMRDEGs in the PPI network were selected as hub genes. GeneMANIA (http://genemania.org) (Franz et al., 2018), a website for analyzing gene functions in gene lists and prioritizing genes for functional assays, was used to construct a PPI network of the hub genes.

### 2.6 mRNA-miRNA and mRNA-transcription factor interaction network

miRNA plays a crucial role in biological development processes by regulating a wide range of target genes. However, it can also be regulated by numerous other miRNAs. The miRDB database (http://mirdb.org) (Chen and Wang, 2020), which predicts miRNA target genes and their functional annotations, is used to predict miRNAs and hub genes that interact with miRNA. The mRNA-miRNA interaction network was constructed using data with a Target Score greater than 90.

TF is the intersection point of multiple signalling pathways in eukaryotic cells and controls mRNA expression (Papavassiliou and Papavassiliou, 2016). Millions of transcription factor binding sites (TFBSs) and TF-miRNA regulatory interactions are available in the CHIPBase database (Version 3.0) (https://rnasysu.com/chipbase3/index.php), which also includes high-throughput sequencing ChIP-Seq data (Huang et al., 2023; Yang et al., 2013). The hTFtarget database (https://guolab.wchscu.cn/hTFtarget/) (Zhang Q. et al., 2020) offers a wealth of human TF targets and information on epigenetic modifications, which we used to predict TF-target regulations. We used the CHIPBase and hTFtarget databases to identify TFs regulated by hub genes and to map mRNA-TF interaction networks. The mRNA-miRNA and mRNA-TF interaction networks were visualized by using Cytoscape.

### 2.7 Immune infiltration analysis

ssGSEA was utilized to categorize IPF patients from GSE24206 and GSE110147 into clusters with diverse immune cell infiltrations, identifying each type of infiltrating immune cell, like CD8^+^ T cells, macrophages, dendritic cells, and others. We conducted the ssGSEA employing the GSVA package (Version 1.50.0) (Hänzelmann et al., 2013) to thoroughly evaluate the immunologic attributes of every sample included in the study. Boxplots were employed to display the variance between different groups (IPF/Control) from GSE24206 and GSE110147 in terms of immune cell infiltration. The association between distinct immune cells in GSE24206 and GSE110147 was determined using the Spearman algorithm, and this was exhibited utilizing the ggplot2 package. We then combined the gene expression matrix of GSE24206 and GSE110147 to calculate the relationship between EMRDEGs and immune cells in various groups (IPF/Control). The ggplot2 package was adopted to produce dot plots.

CIBERSORT, a versatile computational approach, is utilized to estimate cell fractions from the gene expression profile of bulk tissues (GEPs) (Chen et al., 2018; Newman et al., 2015). This technique allows the estimation of immune composition in solid tissues. We applied the LM22 gene signature matrix to quantify the fraction of immune cells using the CIBERSORT algorithm. We selected data that had an immune cell enrichment score greater than zero. Eventually, we compiled the immune cell infiltration matrix, which was showcased using a group comparison graph. We employed stacked column charts to depict the composition profiles of differences in immune cell infiltration between distinct groups (IPF/Control) in studies GSE24206 and GSE110147. The Spearman algorithm was used to calculate the correlations of individual immune cells in these studies, which was demonstrated by using the ggplot2 package. We explored the relationship between immune cells and EMRDEGs in the GSE24206 and GSE110147 studies, creating dot plots with the help of the ggplot2 package.

### 2.8 Statistical analysis

We processed and analyzed our study’s data using R software (version 4.2.2). We compared variables between different groups (IPF/Control) in the datasets, examining the statistical significance of normally distributed data with Student’s t-test and non-normally distributed data with the Mann-Whitney-Wilcoxon test. Unless specifically annotated otherwise, we derived all findings from Spearman correlation analysis, considering P < 0.05 as statistically significant.

## 3 Results

### 3.1 Analysis of differentially expressed genes associated with idiopathic pulmonary fibrosis

First, we utilized the SVA package (Version 3.50.0) and the limma package to eliminate batch effects and standardize GSE24206 and GSE110147 (Supplementary Figure S2). GSE24206 incorporated 17 IPF samples and six healthy adult control samples. GSE110147 encompassed 22 IPF patients and 11 control samples. The results indicated that the batch effects of GSE24206 and GSE110147 were essentially eliminated.

**FIGURE 1:**
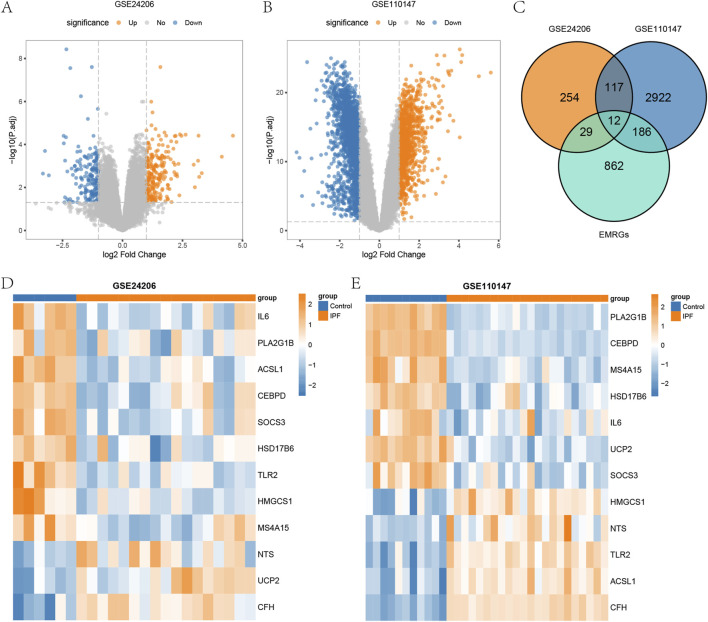
Differential analysis of datasets GSE24206 and GSE110147. **(A)** Volcano plot of DEGs between IPF and Control in GSE24206. **(B)** Volcano plot of DEGs between IPF and Control in GSE110147. **(C)** Venn diagram of DEGs and EMRGs of GSE24206 and GSE110147. **(D)** Heatmap of EMRDEGs in GSE24206. **(E)** Heatmap of EMRDEGs in GSE110147. DEGs, differentially expressed genes; IPF, Idiopathic pulmonary fibrosis; Control, control group; EMRGs, energy metabolism related genes; EMRDEGs, energy metabolism related differentially expressed genes.

We then analyzed the differences in gene expression between the IPF group and the control group, conducting differential gene expression analysis in the GSE24206 and GSE110147 datasets using the limma package. We identified 21,655 DEGs in GSE24206, of which 412 DEGs (with |logFC| >1 and P < 0.05) were found, consisting of 227 upregulated genes and 185 downregulated genes. The GSE110147 dataset contained 21,408 DEGs, and we identified 3,237 DEGs (with |logFC| > 1 and P < 0.05). These consisted of 1,179 upregulated genes and 2,058 downregulated genes. We visualized the expression of DEGs in GSE24206 and GSE110147 using a volcano plot (Figures 1A,B). To obtain the energy metabolism-related DEGs (EMRDEGs) in IPF, we intersected DEGs from both datasets and the EMRGs. We subsequently obtained 12 EMRDEGs (*ACSL1*, *CEBPD*, *CFH*, *HMGCS1*, *HSD17B6*, *IL6*, *MS4A15*, *NTS*, *PLA2G1B*, *SOCS3*, *TLR2*, *UCP2*) and visualized them using a Venn diagram (Figure 1C). Using the pheatmap package, we created heatmaps to display the differential expression of EMRDEGs in GSE24206 and GSE110147 (Figures 1D,E). The figures indicated significant differences in EMRDEGs between GSE24206 (Figure 1D) and GSE110147 (Figure 1E).

### 3.2 Gene ontology and Kyoto Encyclopedia of Genes and Genomes enrichment analyses of energy metabolism-related differentially expressed genes

**FIGURE 2:**
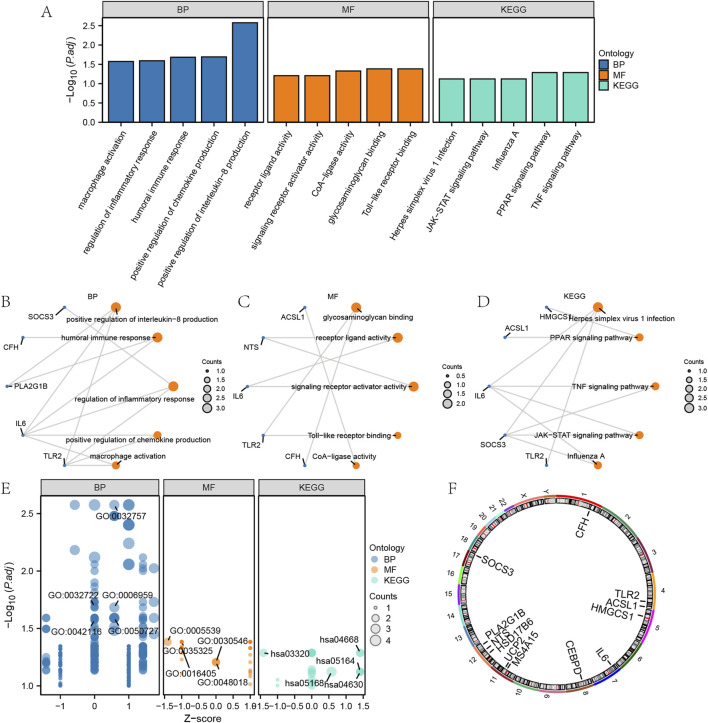
GO and KEGG enrichment analysis of EMRDEGs. **(A)** GO and KEGG enrichment analysis results of EMRDEGs are shown in bar graph. **(B–C)** Circular network diagram of BP **(B)** and MF **(C)** of GO enrichment analysis of EMRDEGs. **(D)** Ring network diagram of KEGG enrichment analysis of EMRDEGs. **(E)** Bubble plot of GO and KEGG enrichment analysis of EMRDEGs. **(F)** Chromosomal localization map of EMRDEGs. In bubble plot **(A)**, the abscissa is the GO terms and the ordinate represents the P values of GO terms. In the Ring network diagram **(B–D)**, blue dots represent specific genes and orange dots represent specific pathways. In bubble plot **(E)**, blue dots represent BP, orange dots represent MF and cyan dots represent KEGG pathways. GO, Gene ontology enrichment analysis; BP, biological process; MF, molecular function; KEGG, Kyoto Encyclopedia of Genes and Genome pathway enrichment analysis; EMRDEGs: Energy metabolism related differentially expressed genes.

We conducted GO and KEGG enrichment analyses of EMRDEGs to assess BP, MF, CC, and pathways (Supplementary Figure S3). Data with FDR <0.05, as well as P < 0.05, were considered statistically significant. In the BP category, EMRDEGs were primarily enriched in “positive regulation of interleukin-8 production”, “humoral immune response” and “regulation of inflammatory response”. In the MF category, EMRDEGs were primarily enriched in “glycosaminoglycan binding”, “receptor ligand activity” and “signalling receptor activator activity”. In the KEGG enrichment analysis, EMRDEGs were primarily enriched in “Herpes simplex virus 1 infection”, “PPAR signalling pathway” and “TNF signalling pathway”. The results of the GO and KEGG enrichment analyses were displayed in the form of bar graphs and ring network diagrams (Figures 2A–D). Next, we utilized the |logFC| value from the previous GSE24206 enrichment analysis to calculate the corresponding Z-score for each molecule, visualizing them in a bubble plot (Figure 2E). According to Figure 2E, we deduced that EMRDEGs were primarily enriched in the BP category.

To investigate the positioning of the EMRDEGs on human chromosomes, this study further annotated the location of the EMRDEGs utilizing the RCircos package (Version 1.2.2) (Figure 2F). Based on Figure 2F, these EMRDEGs primarily resided on chromosomes 1, 4, 8, 11, 12, and 17. Three of these were solely distributed on chromosome 12, suggesting a close relationship at the genome level.

### 3.3 Gene set enrichment analysis of GSE24206 and GSE110147

**FIGURE 3:**
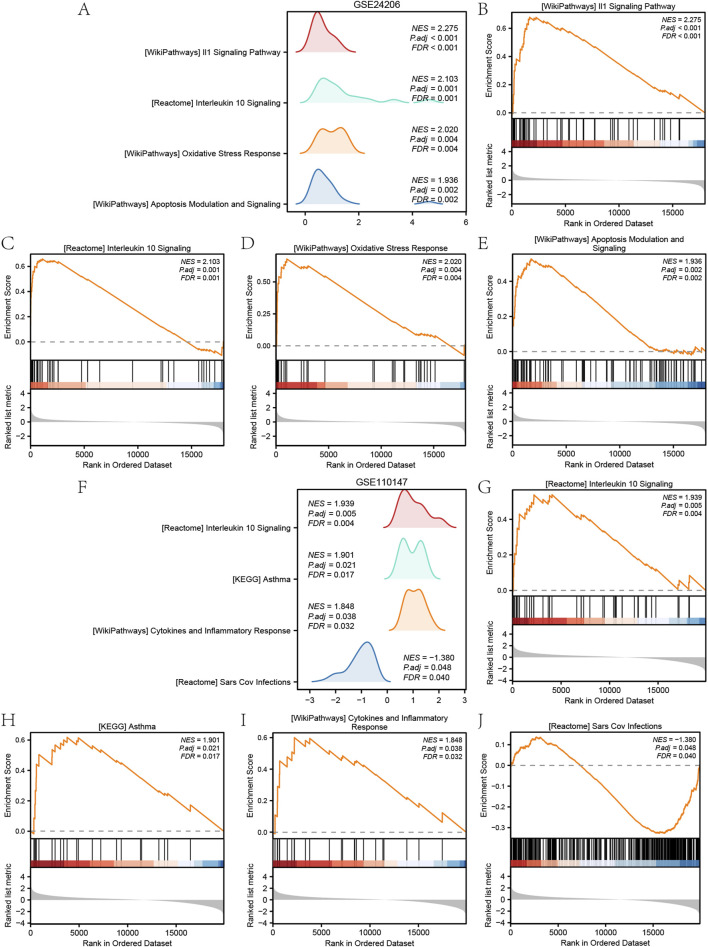
GSEA results of GSE24206 and GSE110147. **(A)** Main biological pathways of GSEA of GSE24206. **(B–E)** Genes in GSE24206 were significantly enriched in WP_IL1_SIGNALING_PATHWAY **(B)**, REACTOME_INTERLEUKIN_10_SIGNALING **(C)**, WP_OXIDATIVE_STRESS_RESPONSE **(D)** and WP_APOPTOSIS_MODULATION_AND_SIGNALING **(E)**. **(F)** Four main biological pathways of GSEA analysis of GSE110147. **(G–J)** Genes in GSE110147 were significantly enriched in REACTOME_INTERLEUKIN_10_SIGNALING **(G)**, KEGG_ASTHMA **(H)**, WP_CYTOKINES_AND_INFLAMMATORY_RESPONSE **(I)** and REACTOME_SARS_COV_INFECTIONS **(J)**. IPF, Idiopathic pulmonary fibrosis. GSEA, Gene Set Enrichment Analysis.

To determine the effect of gene expression levels in different groups (IPF/Control) in GSE24206 and GSE110147 on the pathological progression of IPF, GSEA was used to assess the expression levels of all genes, including BP, CC, and MF. The assessment was performed in different groups (IPF/Control) of GSE24206 using the selection criteria of P and FDR <0.05. The GSEA results for GSE24206, presented in ridgeline plots (Figure 3A), showed significant enrichment of genes in the IL-1 signalling pathway (Figure 3B), interleukin-10 signalling (Figure 3C), oxidative stress response (Figure 3D), apoptosis modulation signalling (Figure 3E), and other pathways (Supplementary Table S4) between different groups (IPF/Control).

Similarly, in GSE110147, genes between the different groups (IPF/Control) were significantly enriched in interleukin-10 signalling (Figure 3G), asthma (), cytokines and inflammatory response (Figure 3I), SARS-CoV infections (Figure 3J), and other pathways (Supplementary Table S5). The GSEA results of GSE110147 were also depicted in ridgeline plots (Figure 3F).

### 3.4 Protein-protein interaction network, mRNA-miRNA and mRNA-TF interaction network

**FIGURE 4:**
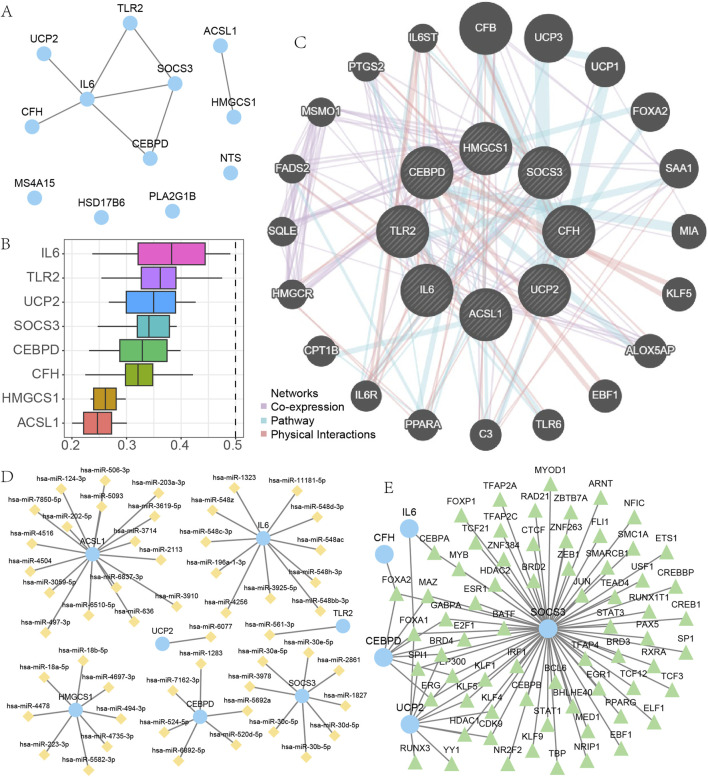
The PPI, mRNA-miRNA and mRNA-TF interaction network. **(A)** PPI network of EMRDEGs. **(B)** Functional analysis of hub genes. **(C)** The results of GeneMANIA database analysis of hub genes. **(D–E)** mRNA-miRNAs interaction network of hub genes **(D)**, mRNA-TF interaction network of hub genes **(E)**. EMRDEGs: Energy metabolism related differentially expressed genes; PPI network, Protein-protein interaction network; TF, Transcription factors.

The STRING database was utilized to examine EMRDEGs and construct a PPI network (medium confidence: 0.400) (Figure 4A). The eight connected nodes were identified as hub genes (*ACSL1*, *CEBPD*, *CFH*, *HMGCS1*, *IL6*, *SOCS3*, *TLR2*, *UCP2*). Subsequently, a functional analysis was conducted to explore the semantic similarity among GO terms, GO term sets, gene products, and gene clusters using the GOSemSim package (Version 2.28.0). This analysis was visualized in boxplots (Figure 4B). As it turned out, among the hub genes, IL6 displayed the highest function similarity value compared with the other hub genes.

We utilized the GeneMANIA database to examine the correlation between hub genes and other genes (Figure 4C). Our results indicated that there were primarily three shared aspects (Co-expression, Pathway, and Physical Interactions) among the hub genes and other genes. Data from the miRDB database concerning mRNA-miRNA was employed to predict miRNA interactions with hub genes; these were then visualized using Cytoscape software (Figure 4D). Within our mRNA-miRNA interaction network, there existed seven hub genes (*ACSL1*, *CEBPD*, *HMGCS1*, *IL6*, *SOCS3*, *TLR2*, *UCP2*), 52 miRNA molecules, and a total of 52 mRNA-miRNAs interaction relationships. The sky-blue circular blocks indicate mRNAs, whereas the yellow rhombus blocks stand for miRNAs. The specific relationships between particular mRNA-miRNAs are presented in Supplementary Table S6.

We searched for and downloaded TFs related to eight hub genes using the CHIPBase and hTFtarget databases. Eventually, we obtained interaction relationships between five hub genes (*CEBPD*, *CFH*, *IL6*, *SOCS3*, *UCP2*) and 67 TFs, and visualized these using Cytoscape software (Figure 4E). Within the mRNA-TF interactions network, the blue circular blocks represented mRNA, while the light green triangular blocks represented TFs. These mRNA - TF interactions are presented in Supplementary Table S7.

### 3.5 Differential expression analysis of EMRDEGs

To determine whether EMRDEGs were differentially expressed, we utilized the signed-rank (Wilcoxon) test to analyze the expression levels of EMRDEGs across different groups (IPF/Control) contained within GSE24206 and GSE110147 (Supplementary Figure S3A,B). Our analysis revealed that all EMRDEGs exhibited highly statistically significant differences in expression levels (P < 0.01).

In GSE24206, the expression of *UCP2* was upregulated in the IPF group, while *TLR2* expression was upregulated in the control group. In GSE110147, *TLR2* and *UCP2* expressions were upregulated in the IPF and control groups, respectively. Among them, *SOCS3* was highly differentially expressed in different groups of both GSE24206 and GSE110147, and this trend was consistent.

We determined the diagnostic value of 8 EMRDEGs using the Receiver Operating Characteristic (ROC) curve (Supplementary Figure S3C–R). In GSE24206, *SOCS3* demonstrated high diagnostic accuracy, while *PLA2G1B* showed a degree of diagnostic accuracy. In GSE110147, *SOCS3* displayed high diagnostic accuracy, and *IL6* presented some diagnostic precision.

### 3.6 Immune infiltration analysis

**FIGURE 5:**
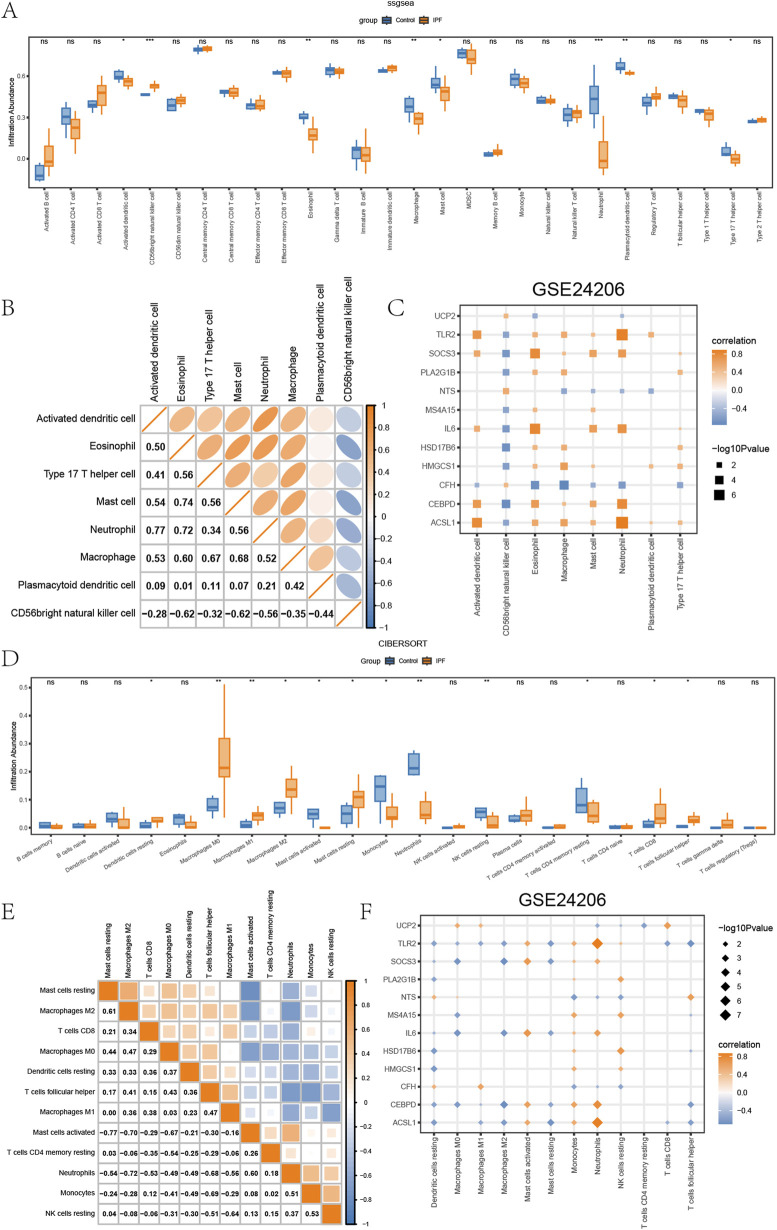
Immune infiltration analysis of GSE24206 **(A)** The ssGSEA results between different groups (IPF/Control) of GSE24206. **(B)** Heatmap of the correlation of the abundance of immune infiltration of 8 types of immune cells in GSE24206. **(C)** Dot plots of the correlation between 8 types of immune cells and EMRDEGs. **(D)** Results of CIBERSORT algorithm of 22 types of immune cells in different groups (IPF/Control) of GSE24206. **(E)** Heatmap of immune cell correlation analysis in GSE24206. **(F)** Dot plots of the association between 18 types of immune cells and EMRDEGs. The symbol ns represents *P* ≥ 0.05, which means not statistically significant. The symbol * represents *P* < 0.05, which means statistically significant; the symbol ** represents *P* < 0.01, which means remarkable statistically significant; the symbol * * * represents *P* < 0.001, which means highly statistically significant. IPF, Idiopathic pulmonary fibrosis. EMRDEGs, Energy metabolism related differentially expressed genes; ssGSEA, single-sample gene set enrichment analysis algorithm.

In examining differences between various groups (IPF/Control) in GSE24206 regarding immune infiltration, we applied ssGSEA to assess the abundance of immune infiltration by 28 diverse kinds of immune cells in DED/Control samples from different groups (IPF/Control) of GSE24206. Subsequently, for the analysis of differences in the infiltration of these 28 varieties of immune cells between the IPF and control groups, we used the Mann-Whitney U test. The results were displayed in boxplots (Figure 5A). According to these findings, the immune infiltration abundance of eight specific types of immune cells showed significant differences (P < 0.05) in the different groups (IPF/Control) of GSE24206. These include activated dendritic cells, CD56bright natural killer cells, Eosinophils, Macrophages, Mast cells, Neutrophils, Plasmacytoid dendritic cells, and Type 17 T helper cells.

Furthermore, we employed Spearman’s correlation to explore the relationship between these eight immune cell types’ infiltration abundance in GSE24206 (Figure 5B). Here, most of them demonstrated positive correlations, except CD56bright natural killer cells. Spearman’s correlation was also used to check the correlation between these eight types of immune cells and EMRDEGs, and the results, exhibited in dot plots (Figure 5C), indicated a significance level of P < 0.05. The data indicated that, despite the presence of negative correlation pairs between these eight immune cell types and EMRDEGs in GSE24206, positive correlation pairs were more prevalent.

Next, we utilized the CIBERSORT algorithm to analyze the infiltration abundance of 22 types of immune cells across GSE24206s different groups (IPF/Control). The results were displayed in boxplots (Figure 5D). The findings revealed that the infiltration abundance of these 22 immune cell types in GSE24206 was not universally 0 and showed a considerable difference between different groups (IPF/Control) (P < 0.05). We turned again to Spearman’s correlation, this time assessing the relative infiltration abundance of 12 types of immune cells and found a significant negative association among these immune cells (Figure 5E). In addition, we used Spearman’s correlation to inspect the relationship between 18 types of immune cells and EMRDEGs (Figure 5F). The results showed that in GSE24206, the number of negative correlation pairs exceeded the number of positive correlation pairs.

**FIGURE 6:**
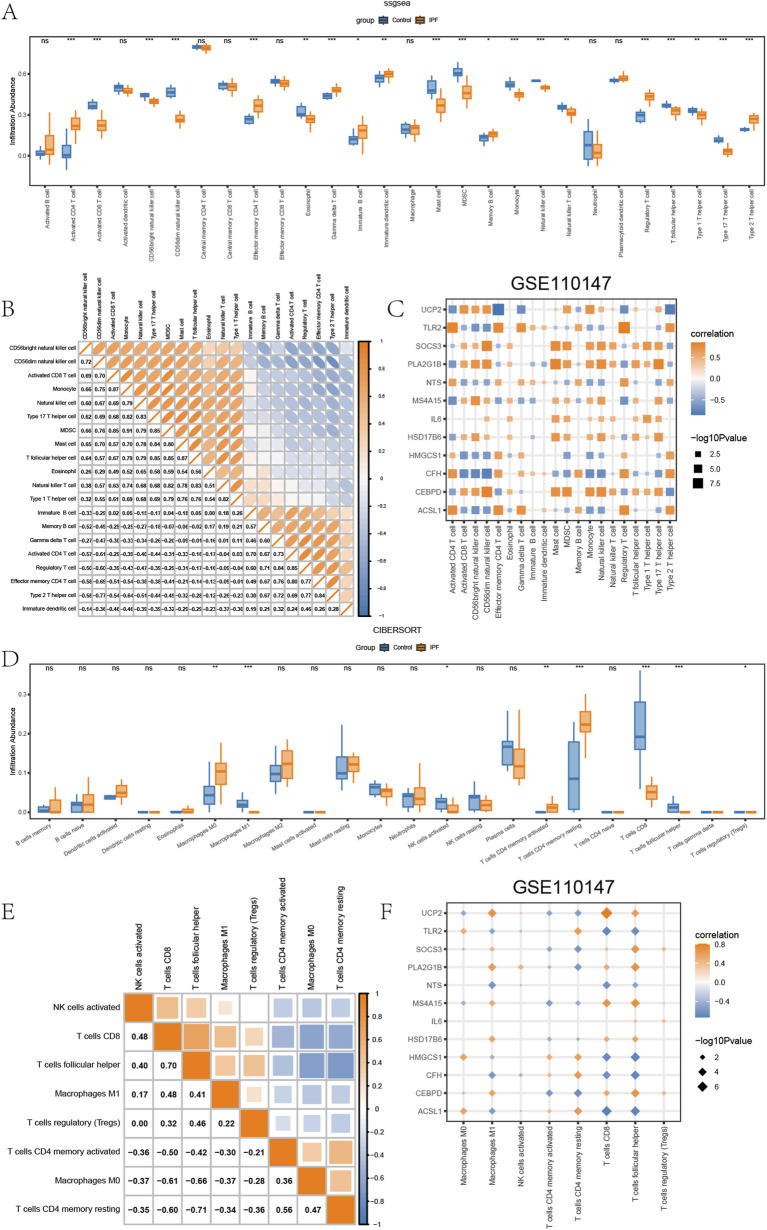
Immune infiltration analysis of GSE110147. **(A)** The ssGSEA results between different groups (IPF/Control) of GSE110147. **(B)** Heatmap of the correlation of the abundance of immune infiltration of 8 types of immune cells in GSE110147. **(C)** Dot plots of the correlation between 20 types of immune cells and EMRDEGs. **(D)** Results of CIBERSORT algorithm of 22 types of immune cells in different groups (IPF/Control) of GSE110147. **(E)** Heatmap of immune cell correlation analysis in GSE110147. **(F)** Dot plots of the association between 8 types of immune cells and EMRDEGs. The symbol ns represents *P* ≥ 0.05, which means not statistically significant. The symbol * represents *P* < 0.05, which means statistically significant; the symbol ** represents *P* < 0.01, which means remarkable statistically significant; the symbol * * * represents *P* < 0.001, which means highly statistically significant. IPF, Idiopathic pulmonary fibrosis. EMRDEGs, Energy metabolism related differentially expressed genes; ssGSEA, single-sample gene set enrichment analysis algorithm.

We utilized the same analysis procedure for GSE110147 as we did for GSE24206. The results are presented in Figures 6A–F. The level of immune infiltration of 20 types of immune cells was significantly different (P < 0.05) for the distinct groups (IPF/Control) within GSE110147. The involved immune cells were activated CD4^+^ T cells, activated CD8^+^ T cells, CD56dim natural killer cells, CD56bright natural killer cells, Effector memory CD4^+^ T cells, Eosinophils, Gamma-delta T cells, Immature dendritic cells, Immature B cells, Mast cells, Myeloid-derived suppressor cells, Memory B cells, Monocytes, Natural killer cells, Natural killer T cells, Regulatory T cells (Tregs), Tfh, Type 1 T helper cells, Type 17 T helper cells, and Type 2 T helper cells. Subsequently, the correlation of the level of immune infiltration of these 20 immune cells in GSE110147 (Figure 6B) demonstrated positive correlations amongst each other. The correlation between these 20 immune cell types and EMRDEGs indicated while there were negative correlation pairings involving eight types of immune cells and EMRDEGs in GSE110147, there were more positive correlation pairs (Figure 6C). The CIBERSORT algorithm results indicated that the level of immune cell infiltration for eight types of immune cells in GSE110147 was not all 0, and there were statistically significant differences between groups (IPF/Control) (P < 0.05) (Figure 6D). Spearman’s correlation was then applied to assess the relative level of immune cell infiltration in these 8 types of immune cells, and a significant negative association was found among these immune cells (Figure 6E). Moreover, using Spearman’s correlation, we analyzed the relationship between these 8 immune cell types and EMRDEGs (Figure 6F). The results revealed that in GSE110147, the number of negative correlation pairs exceeded the number of positive correlation pairs.

## 4 Discussion

This study investigated the role of energy metabolism in IPF. By integrating two independent datasets, GSE24206 and GSE110147, and employing bioinformatics analysis, 12 EMRDEGs were systematically identified. Notably, *HMGCS1*, *MS4A15*, and *PLA2G1B* were newly discovered in the context of IPF. Among these, *HMGCS1* constitutes one of the eight core hub genes (*ACSL1*, *CEBPD*, *CFH*, *HMGCS1*, *IL6*, *SOCS3*, *TLR2*, *UCP2*) warranting further investigation.

*HMGCS1* serves as a critical enzyme in cholesterol synthesis (Chen et al., 2022), which is closely linked to pulmonary surfactant function. In alveolar type II cells, LPS-induced overexpression of *HMGCS1* disrupts pulmonary surfactant homeostasis by dysregulating lipid metabolism, establishing this rate-limiting enzyme in cholesterol biosynthesis as a mechanistic contributor to ARDS-associated pulmonary dysfunction (Chen et al., 2023). The co-occurrence of *HMGCS1* dysregulation, cholesterol accumulation, and surfactant dysfunction in fibrotic lungs raises the possibility that this enzyme contributes to disease progression through lipid metabolic pathways. Recent study suggests that dysregulated cholesterol metabolism mediates abnormal alveolar remodeling and drives pulmonary fibrosis, with surfactant protein C deficiency exacerbating this process by disrupting cholesterol homeostasis (Ruwisch et al., 2020). Additionally, cholesterol-lowering combination therapy alleviated pulmonary inflammation and fibrosis in hypercholesterolemic models through serum cholesterol reduction, directly linking lipid-lowering interventions to suppressed oxidative stress and fibrotic remodeling in the lung, thereby highlighting cholesterol dysregulation as a driver of pulmonary pathology (Seenak et al., 2022). Abnormalities in the pulmonary surfactant system and the formation of deposits are associated with fibroproliferation induced by lung tissue inflammation, primarily due to surfactant dysfunction that promotes extracellular matrix deposition and alveolar space loss, thereby driving fibrosis and honeycomb changes (Beike et al., 2019). The accumulation of alveolar fibrin caused by abnormal cholesterol metabolism and subsequent lung tissue inflammation may represent an intrinsic mechanism by which the *HMGCS1* gene contributes to the pathological progression of IPF. *HMGCS1* acts as an upstream regulator of STAT3 and mediates the proliferation and inflammatory response of psoriatic keratinocytes via the STAT3/IL-23 axis (Chen et al., 2024). Similarly, in cervical cancer, STAT3-miR-223 regulates *HMGCS1* expression, influencing disease progression (Zhang J. et al., 2020). Building on these findings, our study reveals that, in addition to miR-223, miR-18a-5p also functions as a regulatory factor for *HMGCS1*. This finding is supported by prior reports that miR-18a-5p levels decrease in bleomycin-treated alveolar macrophages, where it regulates the TGF-β-Smad2/3 signaling pathway, impacting the epithelial-mesenchymal transition (EMT) of pleural mesothelial cells (Zhang et al., 2017). To further explore *HMGCS1*’s functional network, we utilized the GeneMANIA database, we constructed a gene function network, revealing a potential pathway relationship between *HMGCS1* and PPARα (Kumar et al., 2020; Montaigne et al., 2021). It has been established that PPARs regulate the expression of specific target genes involved in energy and lipid metabolism, adipogenesis, and inflammation (Beigoli et al., 2025). Activation of PPARα inhibits NF-κB transcription and oxidative stress, reducing inflammatory cytokine release (González-Mañán et al., 2017). Furthermore, PPARα effectively suppresses TGF-β1 expression in human lung fibroblasts (HLF) and RAW264.7 cells (Liu et al., 2017). Consistently, mixed PPARα/γ agonists regulate inflammatory cytokines by inhibiting TGF-β1, alleviating liver fibrosis (Yoon et al., 2015). Based on these findings, we propose that the IPF metabolism-related gene *HMGCS1,* regulated by miR-18a-5p, may participate in the pathological process of IPF through STAT3 and PPARα pathways, but it requires further experimental validation.

Another metabolism-related hub gene of interest in the context of IPF is *SOCS3*, which exhibits stable differential expression and high diagnostic accuracy across both datasets, suggesting its potential as a core biomarker for IPF. *SOCS3*, a member of the suppressor of cytokine signaling family, primarily regulates cytokine receptor signaling by inhibiting the JAK/STAT pathway and modulating STAT3 activity. (Liu and Wang, 2022). MiRNA-mediated mRNA degradation and translational inhibition constitute the primary mechanisms regulating *SOCS3* expression (Al-Asadi et al., 2023; Boosani and Agrawal, 2015). Notably, overexpression of miR-30 is observed in glioma stem cells; downregulation of miR-30 reduces *SOCS3* suppression, activating the JAK/STAT3 signaling pathway, thus confirming the regulatory role of the miR-30/*SOCS3*/JAK/STAT3 axis (Che et al., 2015). In line with this, our mRNA-miRNA interaction network predicts that all subtypes of miR-30 (miR-30a-5p, miR-30b-5p, miR-30c-5p, miR-30d-5p, and miR-30e-5p) regulate *SOCS3*. Functionally, the *SOCS* domain inhibits fibronectin and collagen matrix assembly, reduces α-SMA levels, and mitigates matrix deposition in lung fibroblasts and experimental pulmonary fibrosis (Magdaleno et al., 2024). As a negative regulator, *SOCS3* negatively regulates IL-6 via JAK/STAT pathway inhibition (He and Tian, 2021; Qin et al., 2019). Persistent upregulation of *SOCS3* in IPF may reflect compensatory mechanisms to counterbalance chronic immune dysregulation. Our TF-mRNA interaction analysis further reveals that *SOCS3* is associated with hub genes (*IL6*, *CEBPD*, and *UCP2*) through FOXA1 and interacts with *CFH*/*CEBPD* via FOXA2. This positions FOXA as a pivotal transcription factor, suggesting that targeting FOXA could potentially regulate SOCS3 and multiple key genes (*IL6*, *CEBPD, UCP2*, *CFH*). However, while *SOCS3*’s anti-fibrotic roles are well-documented in hepatic, renal, and cardiac fibrosis, its pulmonary-specific mechanisms remain underexplored. To address this gap, studiestargeting the “FOXA-*SOCS3*-JAK/STAT” axis are needed to delineate the diagnostic potential and pathogenic roles in pulmonary fibrosis.

Notably, the diagnostic value of the key gene *IL6* in GSE11014701 aligns with its central position in the PPI network, supporting its dual roles as a functional core and clinical biomarker. Building on this, it is worth investigating whether existing therapies targeting the *IL6*/JAK-STAT pathway (e.g., tocilizumab) influence *SOCS3* overexpression.

Beyond the central roles of *HMGCS1*, *SOCS3*, and *IL6* in IPF pathogenesis, our analysis identifies *ACSL1*, *TLR2*, *UCP2*, *CFH*, and *CEBPD* as synergistic contributors of fibrotic progression through interconnected metabolic-inflammatory crosstalk. *ACSL1*-driven lipotoxicity (Barnhart et al., 2025) primes *TLR2*-dependent inflammatory signaling (Lee et al., 2011), amplifying NF-κB activation (Li et al., 2017; Lin et al., 2021). Concurrently, *UCP2* deficiency exacerbates mitochondrial oxidative stress (Zhu et al., 2023), thereby activating the NLRP3 inflammasome and perpetuating tissue injury (Huang et al., 2024). *CFH* is upregulated in IPF tissues and functions as a core diagnostic biomarker linked to immune dysregulation and extracellular matrix remodeling, implicating complement-mediated fibrotic pathways (Liu et al., 2024), and *CEBPD*-mediated transcriptional reprogramming promotes TGF-β1 overproduction (Lourenço et al., 2020) and lipid metabolic dysregulation (Lai et al., 2017). These interactions coalesce into a self-amplifying cycle wherein lipid overload and oxidative stress sustain inflammatory signaling, which further disrupts metabolic homeostasis, collectively driving sustained fibrotic remodeling. This integrated framework underscores the critical role of metabolic-inflammatory crosstalk in IPF progression.

Building upon this evidence, we next delineated the specific immune contexture shaped by these processes using complementary computational approaches. We employed ssGSEA (single-sample Gene Set Enrichment Analysis) to quantify the activity levels of predefined metabolic and inflammatory gene signatures within individual samples, and CIBERSORT to deconvolve bulk tissue transcriptomes and estimate the relative proportions of infiltrating immune cell subsets. This integrated approach leverages ssGSEA’s capacity to detect coordinated pathway activity and CIBERSORT’s resolution in immune microenvironment characterization, offering a holistic perspective on metabolic-inflammatory dysregulation shaping immune landscapes.

Integrated analysis revealed associations between EMRDEGs and immune dysregulation in IPF. Key immune subsets (e.g., CD56bright NK cells, eosinophils, mast cells, and Th17 cells) exhibited differential infiltration across datasets, linked to inflammatory and fibrotic pathways. Notably, EMRDEGs showed divergent correlations: positive with immune pathway activity (ssGSEA) but negative with immune cell abundance (CIBERSORT). This suggests metabolic reprogramming may perturb immune homeostasis through activity-abundance decoupling, highlighting the complexity of disease-associated immune regulation.

In the GSE24206 dataset, neutrophils were the only immune cell subtype showing consistent positive correlations with EMRDEGs in both ssGSEA (functional activity) and CIBERSORT (cell abundance). This finding implies that neutrophils may orchestrate disease progression through unique molecular pathways, serving as a central hub connecting differential gene expression to immune microenvironment modulation. Specifically, EMRDEGs correlate with neutrophil recruitment and activation, potentially through indirect regulation of chemokine signaling (e.g., modulating upstream pathways of CXCL8) (Ham et al., 2022). Moreover, through pro-inflammatory signaling pathways such as NF-κB or STAT3 (An et al., 2019; Fu et al., 2021), EMRDEGs may drive both neutrophil proliferation (increased abundance) and functional hyperactivation (enhanced activity). Notably, neutrophils can secrete immunosuppressive molecules like arginase and reactive oxygen species (ROS), which may suppress T-cell functionality (Bert et al., 2023).

In stark contrast, analyses of the GSE110147 dataset revealed no cell types with such dual positive correlations, and inconsistencies in immune subpopulations identified by the two methods were observed. This observation suggests that neutrophil activity may be counterregulated by inhibitory signals from other immune cells (e.g., Tregs) (Liu et al., 2025; Tzeng et al., 2022) or microenvironmental factors, thereby obscuring their direct association with EMRDEGs. Collectively, these findings position neutrophils as a context-dependent regulatory target of EMRDEGs, capable of driving synchronized changes in functional activity and cellular abundance in specific disease settings. However, their effects may be masked by competing immune interactions or suppressive microenvironmental cues in alternative contexts, underscoring the necessity for integrative multi-dimensional analyses to unravel these mechanisms.

Future studies should investigate whether targeting EMRDEGs themselves—or their downstream effectors in neutrophil-related pathways—could attenuate fibrotic progression. For example, inhibitors of metabolic enzymes encoded by EMRDEGs (e.g., *HMGCS1*) might disrupt neutrophil bioenergetics, reducing their infiltration and activation.

## 5 Limitations

Our study encountered several limitations. First, the sample size of our data sets was relatively small, which might account for different results from other studies. Second, both data sets lacked crucial information such as treatment details and prognostic clinical parameters of patients. Third, due to limitations in our circumstances, we were unable to verify these genes *in vitro* or *in vivo*. We hope to conduct experimental validation of key genes, focusing on *in vitro* manipulation of candidate genes (e.g., siRNA-mediated knockdown or overexpression) to assess their direct regulatory effects on immune cell recruitment and activation and clarify their contributions to the pathological progression and clinical outcomes of IPF.

## 6 Conclusions

Our analysis identified eight hub genes (*ACSL1*, *CEBPD*, *CFH*, *HMGCS1*, *IL6*, *SOCS3*, *TLR2*, and *UCP2*) associated with IPF progression. Key findings suggest that *HMGCS1* may interact with PPARα signaling to influence fibrotic remodeling, while *SOCS3* potentially coordinates transcriptional regulation of multiple hub genes (*IL6*, *CEBPD*, *UCP2*, *CFH*) through FOXA1/2 and JAK/STAT3 pathway modulation. Immune microenvironment analysis further highlights neutrophils as a central mediator linking hub gene expression to immune dysregulation in IPF. These findings collectively implicate dysregulated energy metabolism and immune microenvironment remodeling as interconnected features of IPF pathogenesis. The prioritized genes (e.g., *HMGCS1*, and *SOCS3*) and their associated pathways (PPARα signaling, FOXA networks, and JAK/STAT3 modulation) represent candidate pathways for mechanistic investigation. While these molecular signatures may inform future biomarker studies, their functional roles and clinical relevance require validation through experimental models. This work provides a foundation for exploring metabolic reprogramming in IPF research.

## Data Availability

The datasets presented in this study can be found in online repositories. The names of the repository/repositories and accession number(s) can be found in the article/Supplementary Material.
