# Supplementary material for: Identification of diagnostic hub genes related to energy metabolism in idiopathic pulmonary fibrosis
Source: Front Mol Biosci. 2025 Jun 26;12:1596364. doi: 10.3389/fmolb.2025.1596364 (PMC12241802; doi:10.3389/fmolb.2025.1596364)
Supplement: Supplementary file 1 [file Table1.docx]

### S1 Table. Idiopathic Pulmonary Fibrosis Dataset Information list.

|  | **GSE24206** | **GSE110147** |
| --- | --- | --- |
| **Platform** | GPL570 | GPL6244 |
| **Experiment type** | Expression profiling by array | Expression profiling by array |
| **Species** | Homo sapiens | Homo sapiens |
| **Tissue** | lung tissue | lung tissue |
| **Samples in Control group** | 6 | 11 |
| **Samples in IPF group** | 17 | 22 |
| **Reference** | Bayesian probit regression model for the diagnosis of pulmonary fibrosis: proof-of-principle. | Comprehensive gene expression profiling identifies distinct and overlapping transcriptional profiles in non-specific interstitial pneumonia and idiopathic pulmonary fibrosis. |

IPF, Idiopathic pulmonary fibrosis.
