# Supplementary material for: Identification of diagnostic hub genes related to energy metabolism in idiopathic pulmonary fibrosis
Source: Front Mol Biosci. 2025 Jun 26;12:1596364. doi: 10.3389/fmolb.2025.1596364 (PMC12241802; doi:10.3389/fmolb.2025.1596364)
Supplement: Supplementary file 4 [file Table7.docx]

### **S7 Table. mRNA-TF interaction network nodes.**

| **mRNA** |  | **TF** |  | **mRNA** |  | **TF** |
| --- | --- | --- | --- | --- | --- | --- |
| **CEBPD** | - | BRD4 |  | SOCS3 | - | KLF9 |
| **CEBPD** | - | E2F1 |  | SOCS3 | - | MAZ |
| **CEBPD** | - | EP300 |  | SOCS3 | - | MED1 |
| **CEBPD** | - | ERG |  | SOCS3 | - | MYB |
| **CEBPD** | - | FOXA1 |  | SOCS3 | - | MYOD1 |
| **CEBPD** | - | FOXA2 |  | SOCS3 | - | NFIC |
| **CEBPD** | - | GABPA |  | SOCS3 | - | NR2F2 |
| **CEBPD** | - | MAZ |  | SOCS3 | - | NRIP1 |
| **CFH** | - | FOXA2 |  | SOCS3 | - | PAX5 |
| **IL6** | - | FOXA1 |  | SOCS3 | - | PPARG |
| **IL6** | - | CEBPA |  | SOCS3 | - | RAD21 |
| **SOCS3** | - | ARNT |  | SOCS3 | - | RUNX1T1 |
| **SOCS3** | - | BATF |  | SOCS3 | - | RXRA |
| **SOCS3** | - | BCL6 |  | SOCS3 | - | SMARCB1 |
| **SOCS3** | - | BHLHE40 |  | SOCS3 | - | SMC1A |
| **SOCS3** | - | BRD2 |  | SOCS3 | - | SP1 |
| **SOCS3** | - | BRD3 |  | SOCS3 | - | SPI1 |
| **SOCS3** | - | BRD4 |  | SOCS3 | - | STAT1 |
| **SOCS3** | - | CDK9 |  | SOCS3 | - | STAT3 |
| **SOCS3** | - | CEBPA |  | SOCS3 | - | TBP |
| **SOCS3** | - | CEBPB |  | SOCS3 | - | TCF12 |
| **SOCS3** | - | CREB1 |  | SOCS3 | - | TCF21 |
| **SOCS3** | - | CREBBP |  | SOCS3 | - | TCF3 |
| **SOCS3** | - | CTCF |  | SOCS3 | - | TEAD4 |
| **SOCS3** | - | E2F1 |  | SOCS3 | - | TFAP2A |
| **SOCS3** | - | EBF1 |  | SOCS3 | - | TFAP2C |
| **SOCS3** | - | EGR1 |  | SOCS3 | - | TFAP4 |
| **SOCS3** | - | ELF1 |  | SOCS3 | - | USF1 |
| **SOCS3** | - | EP300 |  | SOCS3 | - | ZBTB7A |
| **SOCS3** | - | ERG |  | SOCS3 | - | ZEB1 |
| **SOCS3** | - | ESR1 |  | SOCS3 | - | ZNF263 |
| **SOCS3** | - | ETS1 |  | SOCS3 | - | ZNF384 |
| **SOCS3** | - | FLI1 |  | UCP2 | - | BRD4 |
| **SOCS3** | - | FOXA1 |  | UCP2 | - | CDK9 |
| **SOCS3** | - | FOXA2 |  | UCP2 | - | EP300 |
| **SOCS3** | - | FOXP1 |  | UCP2 | - | FOXA1 |
| **SOCS3** | - | GABPA |  | UCP2 | - | HDAC1 |
| **SOCS3** | - | HDAC1 |  | UCP2 | - | KLF1 |
| **SOCS3** | - | HDAC2 |  | UCP2 | - | KLF4 |
| **SOCS3** | - | IRF1 |  | UCP2 | - | KLF5 |
| **SOCS3** | - | JUN |  | UCP2 | - | RUNX3 |
| **SOCS3** | - | KLF1 |  | UCP2 | - | SPI1 |
| **SOCS3** | - | KLF4 |  | UCP2 | - | YY1 |
| **SOCS3** | - | KLF5 |  |  |  |  |

“mRNA”and“TF”represent node；“-”represent edge；TF：Transcription factors.
