# Supplementary material for: Identification of diagnostic hub genes related to energy metabolism in idiopathic pulmonary fibrosis
Source: Front Mol Biosci. 2025 Jun 26;12:1596364. doi: 10.3389/fmolb.2025.1596364 (PMC12241802; doi:10.3389/fmolb.2025.1596364)
Supplement: Supplementary file 6 [file Table5.docx]

### S5 Table. GSEA enrichment analysis results of GSE110147 dataset Control-IPF group genes.

| **Description** | **setSize** | **enrichmentScore** | **NES** | **pvalue** | **p.adjust** | **qvalue** |
| --- | --- | --- | --- | --- | --- | --- |
| **REACTOME_INTERLEUKIN_10_SIGNALING** | 44 | 0.5384 | 1.9385 | 0.0002 | 0.0050 | 0.0042 |
| **KEGG_ASTHMA** | 24 | 0.6179 | 1.9013 | 0.0012 | 0.0206 | 0.0174 |
| **BIOCARTA_IL17_PATHWAY** | 15 | 0.6961 | 1.8898 | 0.0017 | 0.0281 | 0.0236 |
| **WP_ZINC_HOMEOSTASIS** | 36 | 0.5426 | 1.8866 | 0.0011 | 0.0199 | 0.0167 |
| **WP_CYTOKINES_AND_INFLAMMATORY_RESPONSE** | 24 | 0.6004 | 1.8476 | 0.0025 | 0.0375 | 0.0316 |
| **KEGG_ALLOGRAFT_REJECTION** | 25 | 0.5797 | 1.8160 | 0.0024 | 0.0359 | 0.0302 |
| **PID_ALK1_PATHWAY** | 25 | 0.5709 | 1.7885 | 0.0030 | 0.0427 | 0.0359 |
| **WP_FOLATE_METABOLISM** | 60 | 0.4625 | 1.7582 | 0.0018 | 0.0293 | 0.0246 |
| **WP_HEART_DEVELOPMENT** | 44 | 0.4834 | 1.7403 | 0.0023 | 0.0349 | 0.0294 |
| **NABA_SECRETED_FACTORS** | 311 | 0.3069 | 1.5064 | 0.0002 | 0.0061 | 0.0051 |
| **KEGG_CHEMOKINE_SIGNALING_PATHWAY** | 177 | 0.3279 | 1.4897 | 0.0016 | 0.0275 | 0.0231 |
| **KEGG_HUNTINGTONS_DISEASE** | 148 | 0.3389 | 1.4858 | 0.0021 | 0.0335 | 0.0282 |
| **KEGG_ALZHEIMERS_DISEASE** | 136 | 0.3405 | 1.4779 | 0.0021 | 0.0335 | 0.0282 |
| **KEGG_CYTOKINE_CYTOKINE_RECEPTOR_INTERACTION** | 240 | 0.3144 | 1.4726 | 0.0004 | 0.0107 | 0.0090 |
| **REACTOME_SARS_COV_INFECTIONS** | 320 | -0.3281 | -1.3797 | 0.0037 | 0.0478 | 0.0402 |

GSEA: Gene Set Enrichment Analysis; IPF, Idiopathic pulmonary fibrosis.
