# Supplementary material for: Identification of diagnostic hub genes related to energy metabolism in idiopathic pulmonary fibrosis
Source: Front Mol Biosci. 2025 Jun 26;12:1596364. doi: 10.3389/fmolb.2025.1596364 (PMC12241802; doi:10.3389/fmolb.2025.1596364)
Supplement: Supplementary file 7 [file Table3.docx]

### S3 Table. GO and KEGG enrichment analysis results of EMRDEGs.

| **ONTOLOGY** | **ID** | **Description** | **GeneRatio** | **BgRatio** | **pvalue** | **p.adjust** | **qvalue** |
| --- | --- | --- | --- | --- | --- | --- | --- |
| **BP** | GO:0032757 | positive regulation of interleukin-8 production | 3/12 | 62/18800 | 7.36E-06 | 2.66E-03 | 1.16E-03 |
| **BP** | GO:0006959 | humoral immune response | 3/12 | 317/18800 | 9.33E-04 | 2.07E-02 | 9.08E-03 |
| **BP** | GO:0050727 | regulation of inflammatory response | 3/12 | 394/18800 | 1.75E-03 | 2.56E-02 | 1.12E-02 |
| **BP** | GO:0032722 | positive regulation of chemokine production | 2/12 | 70/18800 | 8.80E-04 | 2.03E-02 | 8.88E-03 |
| **BP** | GO:0042116 | macrophage activation | 2/12 | 106/18800 | 2.00E-03 | 2.67E-02 | 1.17E-02 |
| **MF** | GO:0005539 | glycosaminoglycan binding | 2/11 | 234/18410 | 8.20E-03 | 4.14E-02 | 1.72E-02 |
| **MF** | GO:0048018 | receptor ligand activity | 2/11 | 489/18410 | 3.30E-02 | 6.22E-02 | 2.58E-02 |
| **MF** | GO:0030546 | signaling receptor activator activity | 2/11 | 496/18410 | 3.39E-02 | 6.22E-02 | 2.58E-02 |
| **MF** | GO:0035325 | Toll-like receptor binding | 1/11 | 12/18410 | 7.15E-03 | 4.14E-02 | 1.72E-02 |
| **MF** | GO:0016405 | CoA-ligase activity | 1/11 | 27/18410 | 1.60E-02 | 4.69E-02 | 1.94E-02 |
| **KEGG** | hsa05168 | Herpes simplex virus 1 infection | 3/9 | 495/8164 | 1.41E-02 | 7.55E-02 | 4.94E-02 |
| **KEGG** | hsa03320 | PPAR signaling pathway | 2/9 | 75/8164 | 2.88E-03 | 5.16E-02 | 3.38E-02 |
| **KEGG** | hsa04668 | TNF signaling pathway | 2/9 | 112/8164 | 6.31E-03 | 5.16E-02 | 3.38E-02 |
| **KEGG** | hsa04630 | JAK-STAT signaling pathway | 2/9 | 162/8164 | 1.29E-02 | 7.55E-02 | 4.94E-02 |
| **KEGG** | hsa05164 | Influenza A | 2/9 | 171/8164 | 1.43E-02 | 7.55E-02 | 4.94E-02 |

GO, Gene Ontology; BP, biological process; CC, cellular component; MF, molecular function; KEGG: Kyoto Encyclopedia of Genes and Genomes; EMRDEGs: Energy metabolism related differentially expressed genes.
