# Supplementary material for: Identification of diagnostic hub genes related to energy metabolism in idiopathic pulmonary fibrosis
Source: Front Mol Biosci. 2025 Jun 26;12:1596364. doi: 10.3389/fmolb.2025.1596364 (PMC12241802; doi:10.3389/fmolb.2025.1596364)
Supplement: Supplementary file 9 [file Table4.docx]

### S4 Table. GSEA enrichment analysis results of GSE24206 dataset Control-IPF group genes.

| **Description** | **setSize** | **enrichmentScore** | **NES** | **pvalue** | **p.adjust** | **qvalue** |
| --- | --- | --- | --- | --- | --- | --- |
| **WP_IL1_SIGNALING_PATHWAY** | 53 | 0.67901 | 2.27480 | 0.00000 | 0.00004 | 0.00004 |
| **PID_IL6_7_PATHWAY** | 47 | 0.64933 | 2.10614 | 0.00001 | 0.00050 | 0.00041 |
| **REACTOME_INTERLEUKIN_10_SIGNALING** | 43 | 0.65941 | 2.10278 | 0.00003 | 0.00149 | 0.00125 |
| **WP_OXIDATIVE_STRESS_RESPONSE** | 33 | 0.67881 | 2.01994 | 0.00015 | 0.00435 | 0.00363 |
| **WP_APOPTOSIS_MODULATION_AND_SIGNALING** | 84 | 0.52773 | 1.93597 | 0.00006 | 0.00202 | 0.00169 |
| **WP_TH17_CELL_DIFFERENTIATION_PATHWAY** | 66 | 0.55014 | 1.92170 | 0.00016 | 0.00437 | 0.00364 |
| **PID_TCR_CALCIUM_PATHWAY** | 27 | 0.65619 | 1.91084 | 0.00174 | 0.02753 | 0.02296 |
| **KEGG_NITROGEN_METABOLISM** | 22 | 0.68876 | 1.90599 | 0.00124 | 0.02108 | 0.01758 |
| **WP_IL3_SIGNALING_PATHWAY** | 49 | 0.57817 | 1.90563 | 0.00065 | 0.01292 | 0.01078 |
| **WP_ANGIOGENESIS** | 24 | 0.66497 | 1.88945 | 0.00216 | 0.03156 | 0.02632 |
| **PID_CD40_PATHWAY** | 31 | 0.64317 | 1.88428 | 0.00067 | 0.01317 | 0.01098 |
| **PID_PDGFRA_PATHWAY** | 22 | 0.67334 | 1.86333 | 0.00196 | 0.02981 | 0.02486 |
| **REACTOME_SIGNALING_BY_NTRKS** | 132 | 0.47330 | 1.85812 | 0.00002 | 0.00093 | 0.00078 |
| **WP_COMPLEMENT_SYSTEM** | 91 | 0.49276 | 1.84393 | 0.00016 | 0.00437 | 0.00364 |
| **WP_NRF2ARE_REGULATION** | 22 | 0.65833 | 1.82180 | 0.00332 | 0.04332 | 0.03612 |

GSEA, Gene Set Enrichment Analysis; IPF, Idiopathic pulmonary fibrosis.
