# Supplementary material for: Identification of diagnostic hub genes related to energy metabolism in idiopathic pulmonary fibrosis
Source: Front Mol Biosci. 2025 Jun 26;12:1596364. doi: 10.3389/fmolb.2025.1596364 (PMC12241802; doi:10.3389/fmolb.2025.1596364)
Supplement: Supplementary file 10 [file Table6.docx]

### S6 Table. mRNA-miRNA interaction network nodes.

| **mRNA** |  | **miRNA** |  | **mRNA** |  | **miRNA** |
| --- | --- | --- | --- | --- | --- | --- |
| **ACSL1** | - | hsa-miR-3714 |  | SOCS3 | - | hsa-miR-1827 |
| **ACSL1** | - | hsa-miR-6837-3p |  | SOCS3 | - | hsa-miR-30e-5p |
| **ACSL1** | - | hsa-miR-2113 |  | SOCS3 | - | hsa-miR-30a-5p |
| **ACSL1** | - | hsa-miR-124-3p |  | SOCS3 | - | hsa-miR-30d-5p |
| **ACSL1** | - | hsa-miR-4504 |  | SOCS3 | - | hsa-miR-30b-5p |
| **ACSL1** | - | hsa-miR-506-3p |  | SOCS3 | - | hsa-miR-2861 |
| **ACSL1** | - | hsa-miR-636 |  | SOCS3 | - | hsa-miR-30c-5p |
| **ACSL1** | - | hsa-miR-5093 |  | SOCS3 | - | hsa-miR-3978 |
| **ACSL1** | - | hsa-miR-3910 |  | IL6 | - | hsa-miR-11181-5p |
| **ACSL1** | - | hsa-miR-203a-3p |  | IL6 | - | hsa-miR-4256 |
| **ACSL1** | - | hsa-miR-6510-5p |  | IL6 | - | hsa-miR-196a-1-3p |
| **ACSL1** | - | hsa-miR-4516 |  | IL6 | - | hsa-miR-548c-3p |
| **ACSL1** | - | hsa-miR-202-5p |  | IL6 | - | hsa-miR-548z |
| **ACSL1** | - | hsa-miR-7850-5p |  | IL6 | - | hsa-miR-548h-3p |
| **ACSL1** | - | hsa-miR-3059-5p |  | IL6 | - | hsa-miR-548bb-3p |
| **ACSL1** | - | hsa-miR-3619-5p |  | IL6 | - | hsa-miR-1323 |
| **ACSL1** | - | hsa-miR-497-3p |  | IL6 | - | hsa-miR-548ac |
| **TLR2** | - | hsa-miR-561-3p |  | IL6 | - | hsa-miR-3925-5p |
| **HMGCS1** | - | hsa-miR-494-3p |  | IL6 | - | hsa-miR-548d-3p |
| **HMGCS1** | - | hsa-miR-18b-5p |  | UCP2 | - | hsa-miR-6077 |
| **HMGCS1** | - | hsa-miR-18a-5p |  | CEBPD | - | hsa-miR-524-5p |
| **HMGCS1** | - | hsa-miR-4735-3p |  | CEBPD | - | hsa-miR-520d-5p |
| **HMGCS1** | - | hsa-miR-5582-3p |  | CEBPD | - | hsa-miR-6892-5p |
| **HMGCS1** | - | hsa-miR-4697-3p |  | CEBPD | - | hsa-miR-7162-3p |
| **HMGCS1** | - | hsa-miR-223-3p |  | CEBPD | - | hsa-miR-1283 |
| **HMGCS1** | - | hsa-miR-4478 |  | CEBPD | - | hsa-miR-5692a |

“mRNA”and“miRNA”represent node；“-”represent edge.
